# Preserving Renal Function without Compromising Oncological Outcomes: A Comparative Study of Partial and Total Nephrectomies in T3 Stage Renal Cell Carcinoma

**DOI:** 10.15586/jkcvhl.v10i4.290

**Published:** 2023-12-26

**Authors:** Ahmed Alasker, Turki Rashed Alnafisah, Mohammad Alghafees, Areez Shafqat, Belal Nedal Sabbah, Abdullah Alhaider, Abdulrahman Alsayyari, Naif Althonayan, Mohammed AlOtaibi, Salman Bin Ofisan, Mohammed Alghamdi, Nasser Albogami, Abdullah Al-Khayal

**Affiliations:** 1 College of Medicine, King Saud bin Abdulaziz University for Health Sciences, Riyadh, Saudi Arabia;; 2 Department of Urology, King Abdulaziz Medical City, Riyadh, Saudi Arabia;; 3 Department of Medicine, King Abdullah International Medical Research Center, Riyadh, Saudi Arabia;; 4 Alfasial University, College of Medicine;; 5 College of Medicine, Prince Sattam Bin Abdulaziz University, Al-Kharj, Saudi Arabia

**Keywords:** Partial nephrectomy, renal cell carcinoma, stage t3, total nephrectomy

## Abstract

The utility of partial nephrectomy (PN) in locally advanced, stage T3 renal cell carcinoma (RCC) is controversial. This retrospective study aimed to review the oncological and functional outcomes of patients with T3a RCC who underwent PN. We included all patients with pT3a stage RCC undergoing either open, laparoscopic, or robotic PN at our center between January 2015 and 2023. A Wilcoxon rank sum test was utilized to compare nephrectomy types (radical nephrectomy [RN] vs PN). Survival analysis was conducted using Kaplan–Meier plots and a log-rank test. P-value < 0.05 indicated statistical significance. There were no significant differences in demographic characteristics between the RN and PN groups, except age (53.0 vs 6.5, respectively; P = 0.012) and body mass index (28.7 vs 34.3, respectively; P = 0.020). Furthermore, there were also no significant differences in the rates of local recurrence (P = 0.597), metastatic progression (P = 0.129), and chemotherapy use (P = 0.367) between nephrectomy types. Patient survival did not differ significantly based on the type of nephrectomy (log-rank P-value = 0.852). Together, our findings indicated that PN and RN yield near-equivalent oncological outcomes in terms of local recurrence, metastasis, and overall survival rates among pT3a RCC patients during a nearly 3-year follow-up period.

## Introduction

Radical nephrectomy (RN) has traditionally been the gold standard treatment of localized renal cell carcinoma (RCC), offering excellent recurrence-free survival, cancer-specific survival, and overall survival (1). However, recent advancements in surgical techniques have prompted a paradigm shift toward renal preservation, giving rise to partial nephrectomy (PN) to mitigate the development of chronic kidney disease (CKD), reduce cardiovascular morbidity and mortality, and improve patient quality of life, while offering comparable oncological outcomes (2–4).

Partial nephrectomy (PN) is now the new standard of care for renal tumors classified as cT1a (less than 4 cm in size) because it delivers equivalent oncological outcomes to RN, while providing better results in terms of renal function and quality of life (5–7). Furthermore, when technically feasible, PN is favored over RN for the treatment of T1b tumors (4–7 cm), as numerous studies have established no significant differences in cancer-specific survival between PN and RN with better renal preservation in the former (8–10). Recent investigations have begun to shed light on the potential of PN in achieving equivalent oncological outcomes to RN in the treatment of stage T2 RCC, while reducing perioperative morbidity and better maintaining renal function (11–13).

However, the utility of PN in locally advanced stage T3 RCC is controversial. This study reviews the oncological and functional outcomes of patients with T3a RCC (based on the American Joint Cancer Committee 2010 TNM staging criteria) who underwent PN.

## Methods

This retrospective study included patients with pT3a stage RCC who underwent either open, laparoscopic, or robotic PN. Data was collected from the Department of Urology in a tertiary hospital between January 1, 2015 and January 31, 2023. Categorical variables were presented as frequencies and percentages, while numerical variables were expressed as median and interquartile ranges (IQRs). The Wilcoxon rank-sum test was utilized to compare nephrectomy types (radical or partial). Kaplan–Meier plots were used to depict survival curves, and statistical differences in survival were assessed using a log-rank test. P < 0.05 indicated statistical significance.

## Results

A total of147 patients with T3 stage tumors underwent radical and partial nephrectomies (130 and 17 patients, respectively). The median (IQR) age of patients was 63.0 (52.0 and 75.0, respectively) years, with a median body mass index (BMI) of 29.1 (25.0 and 33.6, respectively) kg/m^2^. Most patients were males (n = 90/147; 61.2%) and had no significant medical history before RCC diagnosis (n = 116/147; 78.9%). There were no significant differences in the demographic characteristics between PN and RN groups, except the age (53.0 vs 6.5, respectively; P = 0.012) and BMI (34.3 vs 28.7, respectively; P = 0.020).

**Figure 1:**
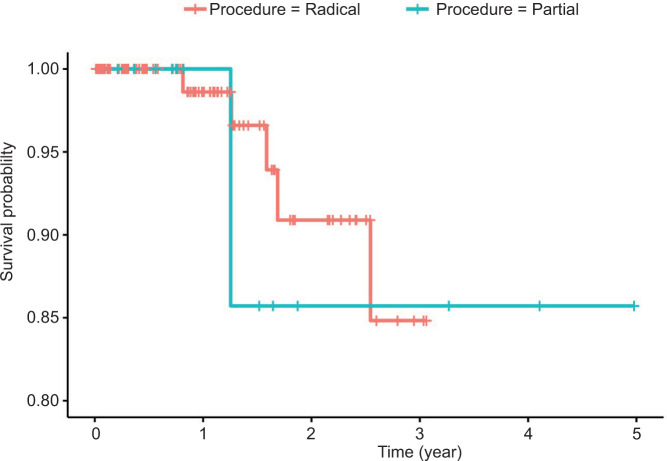
A Kaplan–Meier plot depicting survival curves across types of nephrectomies among patients with T3 RCC (n = 147).

The overall local recurrence rate was 5.7%, and the metastatic progression rate was 14.9%. However, there were no significant differences in the rates of local recurrence (P = 0.597), metastatic progression (P = 0.129), and chemotherapy use (P = 0.367) between nephrectomy types (Table 1). Out of the 147 patients, 8 (5.4%) died. Patients’ survival did not differ significantly based on the type of nephrectomy (log-rank P-value = 0.852, Figure 1).

**Table 1:** Demographic characteristics and outcomes of T3 cancer patients who underwent radical or partial nephrectomies.

Parameter	Category	Overall, N = 147	Nephrectomy type		P	Missing
			Radical, N = 130	Partial, N = 17		
Age	Years	63.0 (52.0, 75.0)	65.5 (52.0, 75.0)	53.0 (47.0, 59.0)	0.012	0 (0%)
Height	m	1.7 (1.6, 1.7)	1.7 (1.6, 1.7)	1.6 (1.6, 1.6)	0.290	0 (0%)
Weight	kg	77.0 (67.0, 89.0)	76.0 (66.6, 88.0)	82.4 (78.0, 95.0)	0.061	0 (0%)
BMI	kg/m^2^	29.1 (25.0, 33.6)	28.7 (24.9, 33.0)	34.3 (26.8, 35.6)	0.020	0 (0%)
Gender	Male	90 (61.2%)	81 (62.3%)	9 (52.9%)	0.456	0 (0%)
	Female	57 (38.8%)	49 (37.7%)	8 (47.1%)		
Nationality	Saudi	142 (97.3%)	125 (96.9%)	17 (100.0%)	> 0.999	1 (0.7%)
	Non-Saudi	4 (2.7%)	4 (3.1%)	0 (0.0%)		
Past medical history (renal)	None	116 (78.9%)	106 (81.5%)	10 (58.8%)	0.080	0 (0%)
	Pyeloplasty	0 (0.0%)	0 (0.0%)	0 (0.0%)		
	Past renal surgery	1 (0.7%)	1 (0.8%)	0 (0.0%)		
	Other	30 (20.4%)	23 (17.7%)	7 (41.2%)		
Local recurrence	Yes	8 (5.7%)	8 (6.4%)	0 (0.0%)	0.597	6 (4.1%)
Metastatic progression	Yes	21 (14.9%)	21 (16.8%)	0 (0.0%)	0.129	6 (4.1%)
Chemotherapy	Yes	14 (9.7%)	14 (10.9%)	0 (0.0%)	0.367	3 (2.0%)

## Discussion

Locally advanced RCC, specifically clinical stage T3, is defined as tumor extension beyond the renal capsule into the venous or collecting systems or invasion into the peripheric or renal sinus fat (14). Stage T3 RCC was historically considered an absolute indication for RN, and this remains the standard as per current guidelines (15). Recent studies, however, have challenged this notion, suggesting a place for PN in the management of these lesions. Furthermore, studies have shown that RCC stage of T3 and beyond imparts a significantly greater risk of preoperative CKD (16); therefore, preserving renal function becomes an increasingly important consideration to improve long-term patient outcomes in terms of function and quality of life.

Our study indicates that PN, predominantly performed robotically at our institute, achieves comparable oncological outcomes for clinical stage T3a RCC to RN: we observed statistically insignificant differences in rates of local recurrence rate, metastatic progression, use of chemotherapy, and overall survival during a median follow-up of 31 months. It is worth noting that the age in the PN group was significantly lower than the RN group (53.0 vs 65.5 years, respectively, P = 0.012) and had a higher BMI (34.3 vs 29.1, respectively, P = 0.020).

Despite the sample size of PN patients, our results align with those from other studies. For instance, Andrade et al. compared 140 patients undergoing robot-assisted RN (n = 70) and PN (n = 70), demonstrating similar 3-year cancer-specific survival (95% vs 94%, respectively, P = 0.78) and recurrence-free survival (100% vs 95%, respectively, P = 0.06) rates between the two groups (17). However, patients who underwent PN showed significantly better preservation of renal function (70% vs 86%, respectively, P = 0.06) (17). Similarly, Yim et al. evaluated surgical and oncological outcomes of PN in a cohort of 157 patients with clinical stage T3a renal masses, reporting 5-year recurrence-free survival, cancer-specific survival, and overall survival rates of 82.1, 93.3, and 91.3%, respectively (18). Importantly, 55.4% of the patients maintained greater than 90% of their estimated glomerular filtration rate (eGFR), with a mean change in the eGFR of 7mL/min/1.72 m^2^ in their patient population (18). Other studies investigating the utility of PN in patients clinically upstaged to T3a have also demonstrated statistically insignificant differences in oncological outcomes between PN and RN, with the former offering superior renal preservation (19–21). In the study by Yim et al., 64% of the patients achieved a trifecta (i.e., negative tumor margins, no perioperative complications, and warm ischemia time ≤ 25 min), of which 37.6% achieved an optimal outcome, defined as patients who additionally preserved greater than 90% of their eGFR and had no increase in their CKD stage (18). A multivariate analysis identified significant predictors of failure to achieve a trifecta or optimal outcome, including higher age (OR 1.06, P = 0.002), increased RENAL nephrometry score (OR 1.30, P = 0.035), and an intraoperative blood loss greater than 300 mL (OR 5.96, P = 0.006) (18).

While encouraging, our findings must be interpreted in light of several limitations. Firstly, the inherent limitation of selection bias for single-center studies and information bias for retrospective designs apply. Additionally, our comparison between the PN and RN pT3a RCC patient cohorts was limited to a selected number of variables. We did not account for intraoperative characteristics such as estimated blood loss, which was the most significant factor determining operative success in the study by Yim et al. (18). We also did not compare the RENAL nephrometry scores—which provide insight into the complexity of renal lesions—between the two groups; it is plausible that patients undergoing PN presented with less complex renal lesions, which may have contributed to their favorable outcomes. Lastly, we were unable to conduct a comprehensive analysis of laboratory parameters, including preoperative, postoperative, and long-term renal function, thereby limiting our ability to evaluate the extent of renal preservation achieved with PN in our sample.

However, we did compare intraoperative outcomes and changes in laboratory parameters over time for all RN and PN procedures for renal cancer patients, but regardless of their stage, during the 8-year period. These additional findings are not mentioned in the tabulated results of the present study. We did not observe statistically significant differences in operative time (P = 0.443) and estimated blood loss (P = 0.114) between the RN and PN groups. However, the incidence of blood transfusions (P = 0.033) and duration of hospital stay (P = 0.004) was significantly lower in the PN group. An analysis of variance (ANOVA) was perfomed to assess the changes in renal function over type for either revealed significantly lower blood urea nitrogen (BUN) in the PN group at three distinct timepoints—preoperative, 3–6 months and 6–12 months—with no significant differences in eGFR or serum creatinine. Nevertheless, admittedly, these differences may not hold in a subgroup analysis of our T3 renal cancer patients.

## Conclusions

Our study suggests that PN and RN yield near-equivalent oncological outcomes in terms of local recurrence, metastasis, and overall survival rates among pT3a RCC patients during a nearly 3-year follow-up period. While our study does provide indirect evidence for comparable operative outcomes between PN and RN and better renal function preservation in the PN group, the lack of a subgroup analysis among pT3a patients between PN and RN groups means that we cannot positively affirm such conclusions. Only future comparative observational and randomized studies with larger sample sizes, longer follow-up times, and more in-depth comparisons between PN and RN groups across different procedure types (open, laparoscopic, robotic) will provide a better indication of the role of PN in the surgical management of patients with locally advanced, stage T3 renal cancer.
